# Herpes simplex virus dissemination with necrotizing hepatitis following Descemet membrane endothelial keratoplasty

**DOI:** 10.1186/s12879-023-08414-6

**Published:** 2023-07-12

**Authors:** Ahad Azeem, Brandon Baartman, Christopher D. Conrady, Jeffery L. Meier, Rima El-Herte

**Affiliations:** 1 Department of Medicine, Creighton University School of Medicine and CHI Health, Omaha, NE 68105 USA; 2 Vance Thompson Vision, Omaha, NE 68137 USA; 3 Departments of Ophthalmology, Pathology, and Microbiology, Medical Center, University of Nebraska, Omaha, NE 68198 USA; 4 Iowa City Veterans Affairs Health Care System, Iowa City, IA 52246 USA; 5 Division of Infectious Diseases, Department of Internal Medicine, Department of Epidemiology, College of Public Health, University of Iowa Carver College of Medicine, Iowa City, IA 52242 USA; 6 Division of Infectious Diseases, Department of Medicine, Creighton University School of Medicine and CHI Health, Omaha, NE 68124 USA

**Keywords:** Cornea transplant, Corneal transplant infection, Herpes keratitis, Herpes simplex hepatitis, Hemophagocytic lymphohistiocytosis

## Abstract

**Background:**

Corneal transplants are the most common type of transplant and increasing in frequency. Donor cornea tissues are a rare source of herpes simplex virus (HSV) transmission and not routinely tested for presence of HSV. Donor graft-to-recipient transmission typically causes graft failure and anterior uveitis, and extra-ocular HSV disease has not been previously reported. We present a case of HSV transmission from donor cornea tissue that nearly cost the corneal transplant recipient his life.

**Case report:**

An elderly immunocompetent man developed an acute illness 10 days after having donor corneal tissue implanted in a Descemet membrane endothelial keratoplasty (DMEK). He was found to have HSV necrotizing hepatitis per liver biopsy, trilineage cytopenia, rhabdomyolysis, acute kidney failure, altered mental status, early-stage hemophagocytic lymphohistiocytosis (HLH), and donor corneal tissue implant infection resulting in graft failure and anterior uveitis. HSV DNA was detected in cerebral spinal fluid, peripheral blood, explanted donor corneal tissue, and anterior chamber fluid (220 million HSV DNA copies per mL). HSV-1 seroconversion denoted a primary HSV infection, and the patient had no other risk factor for HSV acquisition. Early recognition of HSV dissemination prompting treatment with intravenous acyclovir, as well as a short course of HLH-directed therapy, resolved the systemic illness. Vision was restored to near normal by replacement of the infected corneal graft with new donor DMEK tissue in conjunction with intravitreal foscarnet treatment.

**Conclusion:**

Awareness of the potential risk of donor cornea tissue transmitting HSV and leading to life-threatening HSV disease is paramount to early diagnosis and treatment. The role of donor cornea tissue in HSV transmission and disease merits additional attention and investigation.

## Background

Transplantation of full- or partial-thickness donor cornea is the definitive treatment of many causes of blindness related to corneal pathology. The U.S. Food and Drug Administration (FDA) and the Eye Bank Association of America (EBAA) deem unnecessary the screening of donor cornea for presence of herpes simplex virus (HSV) [1, 2]. HSV transmission by donor cornea tissue is rare, typically resulting in primary graft loss, and has not been associated with viral dissemination and death. In one published series, HSV-1 DNA was detected in 2 of 237 corneas from donors without a history of ocular HSV infection [3]. HSV is a common cause of corneal blindness and can cause recurrent corneal graft loss [4]. After reactivating from latency in neuronal cell bodies in the trigeminal ganglion, HSV travels along peripheral sensory nerve fibers to the cornea to replicate in outer surface epithelial or inner surface endothelial cornea layers [5, 6]. Here, we report the first case of cornea transplant-associated HSV-1 dissemination. This occurred in an elderly immunocompetent male 10 days after transplantation of donor cornea tissue used in a Descemet membrane endothelial keratoplasty (DMEK) and who did not have pre-existing immunity to HSV or another risk factor for HSV acquisition.

## Case presentation

A 73-year-old male was taken to the emergency department by family members in December 2021 because of fever, generalized weakness, and mental slowing that developed over five days. He lives with his wife for over 50 years in the U.S. midwest and was working as an engineer from home. He was not sexually active or in close contact with children or people with illness in recent weeks. He had not traveled, been exposed to animals, or performed outdoor activities during this time. Review of systems were negative for visual changes, neck stiffness, mouth sores, chest pain, cough, shortness of breath, abdominal discomfort, nausea, vomiting, diarrhea, skin rash or sores, genitourinary changes, musculoskeletal pain, joint swelling, stroke-like symptoms, neuropathy symptoms, or loss of balance. The patient has diabetes mellitus type 2 (hemoglobin A1C 6.8%, while on metformin and glimepiride), hypertension, and hypothyroidism that have been well-controlled on a stable medication regimen. He has severe Fuchs dystrophy of the cornea limiting his visual potential for which he had received DMEK in each eye. Six weeks prior to the presenting illness, he had received a DMEK regraft in the right eye secondary to suspected primary graft failure, which healed properly. Subsequently, the patient underwent DMEK on the left eye. A graft exchange in the left eye was required 10 days prior to the presenting illness due to suspected primary graft failure, as there was no evidence of keratouveitis involving the first graft. The second DMEK graft was healing nicely with routine post-operative care that included use of topical and intraocular corticosteroid and topical moxifloxacin. Neither he nor his family relatives had a past-history of a serious infection or a problematic immune system.

In the emergency department, the patient appeared ill and lethargic. Verbal responses to questions were delayed and speech slowed, but he was not disoriented. Temperature was 39.4^o^C, pulse rate 91 beats/min, blood pressure 162/82 mm Hg, and respiratory rate 24 breaths/min, with 95% oxygen saturation on room air. Neck was supple and there was no focal neurological deficit. Complete physical examination was otherwise unremarkable. Laboratory studies showed a white blood cell count (WBC) of 4.3 k/µL (reference range (RR), 4–12 k/µL), with 43% neutrophils, 22% lymphocytes, and 3% monocytes. Hemoglobin (Hgb) 12.3 g/dL (RR, 13.5–17.5 g/dL), platelets 86 k/µL (RR, 140–440 k/µL), serum creatinine (Cr) 1.69 mg/dL (RR, 0.6–1.3 mg/dL), aspartate transaminase (AST) 527 U/L (RR, 10–40 U/L), alanine transaminase (ALT) 427 U/L (RR, 12–78 U/L), alkaline phosphatase (ALP) 58 µ/L (RR, 33–138 µ/L), and total bilirubin of 0.3 mg/dL (RR, 0-1.5 md/dL). Computed tomography of head, chest, abdomen, and pelvis were without significant findings. The nasopharyngeal swab was negative by polymerase chain reaction (PCR) for respiratory syncytial virus, influenza A and B, and SARS-CoV-2. Serum hepatitis A IgM, hepatitis C IgG, hepatitis B surface antigen, and hepatitis B core IgM were negative. Intravenous (IV) vancomycin and piperacillin-tazobactam were started after blood cultures were obtained. The patient was admitted to the hospital.

On day 2 of hospitalization, the fever and mental slowing continued despite broad-spectrum antibiotic treatment and blood cultures that had no growth. The WBC declined to 2.7k/ µL, with 66% neutrophils and 31% lymphocytes. Platelets and Hgb were unchanged. There were increases in Cr to 2.8 mg/dL, AST 2725 U/L, and ALT 1654 U/L. Bilirubin was normal. Additional testing revealed elevated values for lactate dehydrogenase (LDH) 1333 U/L, creatinine phosphokinase 3024 U/L, triglycerides 222 mg/dL, and ferritin > 20,000 ng/mL. The Infectious Disease team was consulted, and IV acyclovir was added with the dose adjusted to renal function. Antibiotics were changed to IV ampicillin, ceftriaxone, and vancomycin until bacterial infection of the central nervous system was excluded and blood cultures had another day of incubation. Magnetic resonance imaging (MRI) of the brain and fluoroscopy-guided lumber puncture were performed. The brain MRI showed several old lacunar infarcts in watershed distribution and no parenchymal or meningeal enhancement. CSF opening pressure was normal. CSF red blood cells were 530 /µL, white cells 2/µL (RR, 0–5/µL), with 40% lymphocytes. CSF protein was 45 mg/dL (RR, 12–60 mg/dL) and glucose 56 mg/dl (RR, 4–75 mg/dL). The BioFire® meningitis/encephalitis panel detected herpes simplex virus 1 (HSV-1). CSF bacterial culture was negative. HIV antigen-antibody screen was negative. A liver biopsy to evaluate the possibility of herpes simplex hepatitis was discussed with the patient who opted to wait and see if the acyclovir would render the invasive test unnecessary. Serum sent out to an external laboratory for testing returned 5 days later with evidence of HSV DNA in the serum by qualitative PCR and absence of HSV-1 IgG and HSV-2 IgG. The IV acyclovir was continued, and the antibacterial drugs were stopped.

**Fig. 1:**
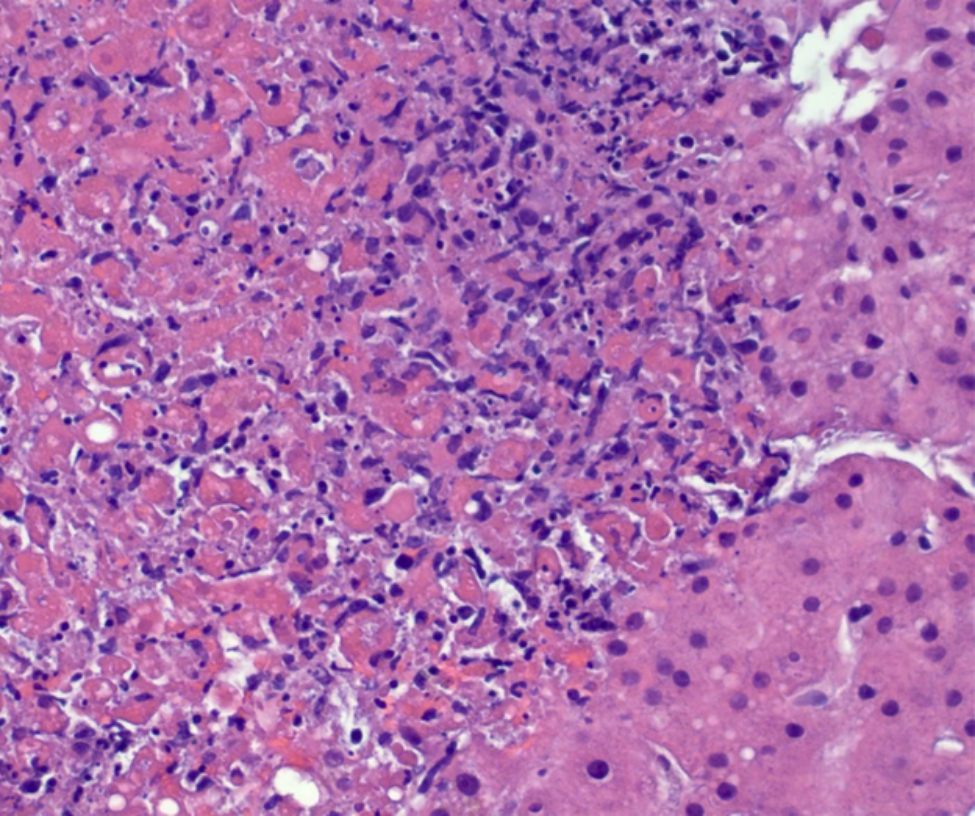
Herpetic non-zonal necrotizing hepatitis. Hematoxylin and eosin stain of liver biopsy tissue

**Fig. 2:**
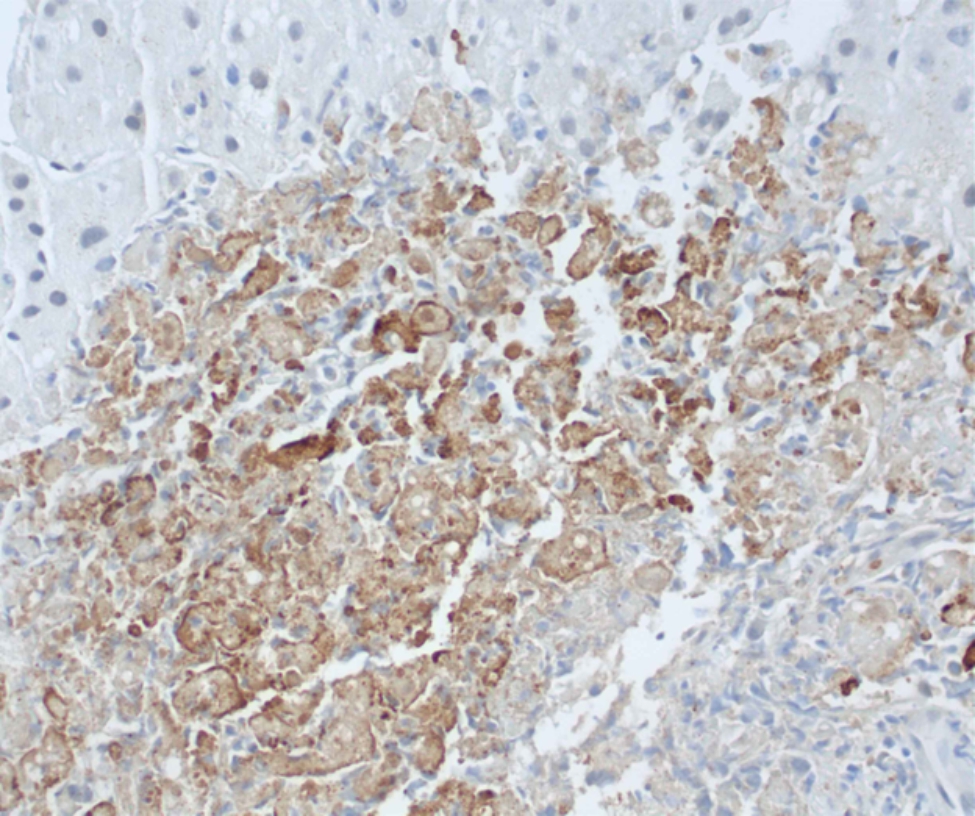
Evidence of HSV-1 in liver biopsy tissue by HSV-1-specific immunohistochemistry (brown color). Hematoxylin (blue) counterstain

On day 5, the fever had resolved but the mental slowing continued. There was an increase in AST of 3719 U/L, ALT 2178 U/L, and Cr 3.93 mg/dL. Total bilirubin remained normal at 0.4 mg/dL. There was a slight decrease in platelets at 76 k/µL and Hgb 11.6 g/dL. His WBC was 2.8k/ µL (55%neutrophils and 30%lymphocytes) and fibrinogen 251 mg/dL. The Hematology-Oncology consultant prescribed him a single dose of etoposide and 26 mg dexamethasone daily for treatment of incipient hemophagocytic lymphohistiocytosis (HLH). Serum interleukin-2 receptor was 7270.7 pg/mL (RR, 175.3-858.2 pg/mL). A percutaneous needle biopsy of the liver was performed to evaluate possibilities of HSV hepatitis, HLH, or both. The biopsy showed herpetic non-zonal necrotizing hepatitis (Fig. 1) that was positive by immunohistochemistry for HSV (Fig. 2) and negative for cytomegalovirus. Kupffer cells were minimally increased and an occasional Kupffer cell exhibited erythrophagocytosis. A bone marrow biopsy was not performed because the patient’s hematologic indices were improving, and he opted to forgo additional invasive tests.

On day 7, the patient’s mental status had returned to normal, but he mentioned that his left eye vision was blurred and had a contact lens that was due for removal. On physical exam, the left eye was not injected. The contact lens was removed at bedside by the Ophthalmology consultant. Examination using proparacaine-fluorescein dye revealed a 2–5 mm central vertical defect of the left cornea without anterior chamber inflammation and was scheduled outpatient follow-up examination by the cornea transplant surgeon. Although the AST and ALT started to improve, decline in kidney function necessitated hemodialysis.

On day 10, the dexamethasone was stopped because the liver transaminases, platelets, and Hgb were improving. On day 15, the patient was discharged from hospital to an inpatient rehabilitation center. At the time of discharge, ALT, AST, WBC, Hgb, and platelets had normalized. Hemodialysis was continued. The IV acyclovir had been given for a total of 15 days followed by oral valacyclovir 1 gram thrice daily that was dose-reduced for hemodialysis (500 mg daily).

On the day of discharge to home from inpatient rehabilitation, an outpatient evaluation by the transplant surgeon noted a partially detached edematous left cornea graft that had failed and 4 + cells in the anterior chamber. The patient was scheduled for graft removal and PCR testing of graft tissue for HSV. Evaluation by Infectious Disease noted the patient had regained strength and functional independence and had improving kidney function that would enable discontinuation of hemodialysis next month. Valacyclovir was continued. Serum HSV-1 IgG was rechecked and was detectable. Two days after discharge to home, examination by the Retina and Uveitis Specialist, including use of optical coherence tomography, revealed anterior keratouveitis and corneal graft failure. The anterior keratouveitis precluded an evaluation of the posterior eye with sufficient clarity to exclude the possibility of HSV infection extending to retina or other posterior segments. Thus, intravitreal foscarnet 2.4 mg/0.1mL was given. PCR analysis of anterior chamber fluid obtained by paracentesis detected HSV-1 DNA at 220 million copies/mL. The patient was started on topical difluprednate 0.05% every 4 h, and once the inflammation in the eye had resolved, the failed corneal DMEK graft was replaced with a new donor graft. The explanted corneal tissue subsequently tested positive for HSV-1 DNA by qualitative PCR. Oral valacyclovir was continued along with topical ophthalmic corticosteroid. The patient’s vision improved, and the new corneal implant fully engrafted. One month later, the patient was evaluated for a possible flare of the anterior uveitis. Another intravitreal dose of foscarnet was given, and oral valacyclovir and topical ophthalmic corticosteroid were continued. Anterior chamber fluid analysis showed an HSV-1 DNA load of 100,000 copies/mL. The uveitis subsequently resolved, and vision remained near the patient’s baseline.

At 11 months after the hospital admission, the patient had returned to who his usual state of health and functioning. The left eye corneal transplant was functioning well, with a left eye visual acuity of 20/40. Twice daily ophthalmic corticosteroid was continued. Serum Cr settled in the 1.6–1.8 mg/dL range. Oral valacyclovir was continued to suppress HSV reactivation infection.

## Discussion and conclusions

**Fig. 3:**
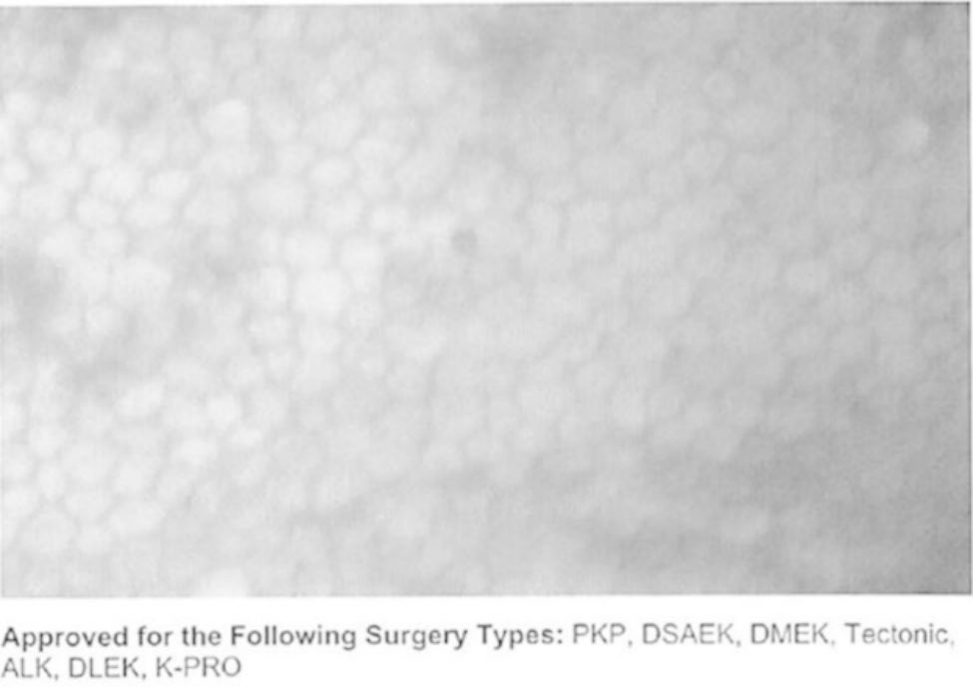
Specular microscopy of the donated ocular tissue suspected to have transmitted the HSV. Healthy hexagonal endothelial cells can be appreciated with little dropout or signs of stress

Donor corneas have been implicated as a source of HSV transmission for over 25 years [7, 8]. This link was later substantiated when molecular DNA fingerprinting techniques showed the HSV DNA genomes in donor corneal tissues before and after transplantation had identical DNA sequences [9, 10]. Reports of HSV transmission by donor corneas generally describe outcomes of graft loss and associated anterior uveitis. The HSV infection is quickly silenced by graft removal and delivery of intraocular and systemic antiviral therapy, which is often followed by use of suppressive dosages of oral antiviral therapy for an extended period. In our patient, the storage of donor cornea in Optosol-GS for 3 days until transplantation and the waiving of testing for HSV are in concordance with FDA and EBAA policies. The donor cornea tissue was obtained from a 66-year-old decedent and death from anoxia to tissue preservation was 6 h. The decedent was a male with chronic obstructive pulmonary diseases and alcohol use disorder who had been resuscitated and placed on life support after found unresponsive in respiratory arrest from alcohol versus carbon monoxide poisoning. While in the intensive care unit, he suffered a cardiac arrest and brain death after which life-support was withdrawn. The EBAA-required screening tests for transmissible diseases (i.e., HIV-1, HIV-2, hepatitis B, hepatitis C, syphilis, HTLV-1, and HTLV-2) were negative, and the tissue was deemed healthy by specular microscopy (Fig. 3). Transplantation of donor cornea from the fellow eye into another recipient was uncomplicated. While we have not proven that the donor cornea was the source of HSV transmission, we believe this is most likely for the following reasons. First, HSV disease appeared 10 days after the DMEK because of a primary HSV-1 infection, as evidenced by HSV-1 seroconversion. Second, no other risk factor for HSV acquisition was identified. This 73-year-old life-long Nebraska resident had been living with his wife of over 50 years, had not been sexually active or exposed to children in preceding weeks, and was avoiding social gatherings to not risk acquiring SARS-CoV-2 infection. His wife never had cold sores or knowledge of having HSV. HSV-1 seroprevalence among adult non-Hispanic white females and males in the U.S. midwest is less than that in other regions of the U.S. and has decreased in recent years [11]. Third, donor corneal Descemet membrane and endothelium layers used in DMEK have been linked to HSV transmission [9, 12, 13], and HSV DNA has been detected in pre-transplant donor corneas given to DMEK recipients who then developed HSV infection causing graft detachment and corneal edema [9, 12, 13]. Lastly, our patient’s corneal HSV infection was limited to the DMEK graft area at the inner corneal surface where it produced anterior chamber inflammation but not retinitis.

The cornea transplant complication of HSV dissemination with multi-organ involvement has not been reported previously. Predisposing factors for the hematogenous spread of HSV in this case include absence of pre-existing immunity against HSV-1 and HSV-2 and possibly an immune response weakened by advanced age and diabetes mellitus. Our patient did not have an inordinate frequency or severity of infections in the past. The resultant HSV hepatitis of hematogenous spread is rare in immunocompetent adults, is rapidly progressive with high mortality, and is oftentimes ascribed to a primary HSV infection during pregnancy [14, 15]. Absence of tell-tale signs of antecedent or concurrent oral or genital herpes is common and contributes to delay in the HSV diagnosis. Presence of HSV DNA in both blood and CSF of our patient is an additional indicator of hematogenous dissemination, although HSV DNAemia can also occur in the absence of HSV infection of viscera [16]. HSV-associated rhabdomyolysis has been described in association with HSV encephalitis [17] and disseminated HSV-induced HLH [18]. The constellation of clinical and laboratory abnormalities in this case yielded a diagnosis of incipient reactive HLH. While severe HSV infection is a known trigger for HLH [19], the observation of hemophagocytosis in the infected liver of our patient is not specific for HLH [20].

Early diagnosis and prompt initiation of antiviral treatment favored a good outcome in this case. Corneal transplantation is the most frequent type of transplantation [21], and the 2019 EBAA statistical report indicates the annual number of these transplantations is increasing. Awareness of the possibility that donor cornea can transmit HSV and bring about HSV hepatitis or dissemination in a vulnerable transplant recipient is paramount to early diagnosis and life-saving treatment.

## Data Availability

All data generated or analyzed during this study are included in this published article.

## Abbreviations

HSV-1: Herpes simplex virus-1
DMEK: Descemet membrane endothelial keratoplasty
HLH: Hemophagocytic lymphohistiocytosis
DNA: Desoxyribonucleic acid
FDA: U.S. Food and Drug Administration
EBAA: Eye Bank Association of America
WBC: White blood cells
SARS-CoV-2: Severe acute respiratory distress syndrome-coronavorus-2
CSF: Cerebrospinal fluid
PCR: Polymerase chain reaction
